# A novel binding pocket in the D2 domain of protein tyrosine phosphatase mu (PTPmu) guides AI screen to identify small molecules that modulate tumour cell adhesion, growth and migration

**DOI:** 10.1111/jcmm.17973

**Published:** 2023-10-20

**Authors:** Kathleen Molyneaux, Christian Laggner, Susann M. Brady‐Kalnay

**Affiliations:** ^1^ Department of Molecular Biology & Microbiology Case Western Reserve University Cleveland Ohio USA; ^2^ Atomwise Inc. San Francisco California USA

**Keywords:** artificial intelligence, cell adhesion, cell–cell adhesion, drug design, glioblastoma, glioma, protein tyrosine phosphatase mu, receptor protein tyrosine phosphatase, tyrosine phosphatase

## Abstract

Approximately 40% of people will get cancer in their lifetime in the US, and 20% are predicted to die from the condition when it is invasive and metastatic. Targeted screening for drugs that interact with proteins that drive cancer cell growth and migration can lead to new therapies. We screened molecular libraries with the AtomNet® AI‐based drug design tool to identify compounds predicted to interact with the cytoplasmic domain of protein tyrosine phosphatase mu. Protein tyrosine phosphatase mu (PTPmu) is proteolytically downregulated in cancers such as glioblastoma generating fragments that stimulate cell survival and migration. Aberrant nuclear localization of PTPmu intracellular fragments drives cancer progression, so we targeted a predicted drug‐binding site between the two cytoplasmic phosphatase domains we termed a D2 binding pocket. The function of the D2 domain is controversial with various proposed regulatory functions, making the D2 domain an attractive target for the development of allosteric drugs. Seventy‐five of the best‐scoring and chemically diverse computational hits predicted to interact with the D2 binding pocket were screened for effects on tumour cell motility and growth in 3D culture as well as in a direct assay for PTPmu‐dependent adhesion. We identified two high‐priority hits that inhibited the migration and glioma cell sphere formation of multiple glioma tumour cell lines as well as aggregation. We also identified one activator of PTPmu‐dependent aggregation, which was able to stimulate cell migration. We propose that the PTPmu D2 binding pocket represents a novel regulatory site and that inhibitors targeting this region may have therapeutic potential for treating cancer.

## 1 INTRODUCTION

Hyperactivation of protein phosphorylation is a hallmark of many cancers and has been shown to drive survival, growth and metastasis. This has stimulated the development of therapeutic agents designed to inhibit the catalytic activity of kinases,^1^ enzymes that phosphorylate specific substrates typically on serine, threonine and/or tyrosine residues. Modulation of phosphatases, which dephosphorylate downstream targets, also results in disease.^2^ However, attempts to target protein tyrosine phosphatases (PTPs) have been considerably less successful than those targeting kinases. Drugs able to interact with the highly charged active sites of tyrosine phosphatases are often not membrane permeable. Also, the active site within the phosphatase domains is highly conserved at the amino acid level meaning targeting agents are likely to be promiscuous.^3^, ^4^ Thus, the phosphatase field has turned its focus towards developing allosteric inhibitors. These agents have the potential to be both more membrane permeable and specific because they may interact with regulatory regions that could be both less charged and more divergent than the catalytic domain.^5^

The protein tyrosine phosphatase mu (PTPμ) is an attractive therapeutic target because its expression is altered in many cancers,^2^, ^6^, ^7^, ^8^, ^9^ with consequent changes in growth and cell motility. PTPμ is known to stimulate neuronal or axonal migration (neurite outgrowth) during development of the nervous system.^10^, ^11^, ^12^, ^13^, ^14^, ^15^ The expression of full‐length PTPμ inhibits cell growth and motility^16^, ^17^ and induces contact inhibition thus functioning as a tumour suppressor. Proteolytic cleavage of PTPμ reverses this effect and generates both extracellular and intracellular fragments that actually stimulate migration of glioblastoma cells and thus function as oncogenes.^18^ There are structural motifs within both the extracellular and intracellular fragments that can be targeted for drug development. We collaborated with Atomwise to identify agents that target the extracellular fragment of PTPμ.^19^ Those chemical agents targeting the PTPμ extracellular domain have also been shown to have therapeutic potential.^19^

Allosteric regions of the intracellular domain of PTPμ can also be exploited for drug development. The intracellular portion of PTPμ consists of a juxtamembrane region^20^ resembling the cytoplasmic region of cadherins^21^ and two highly conserved phosphatase domains, a catalytically active membrane proximal D1 domain and a D2 phosphatase domain. Agents targeting a known regulatory motif termed the ‘wedge’ domain within the juxtamembrane portion of receptor protein tyrosine phosphatase (RPTPs) show therapeutic potential, although the precise role of the wedge domain remains controversial.^22^ Peptides targeting this region in LAR^14^ and PTPσ^23^, ^24^ have been shown to affect downstream signalling, and the PTPσ wedge peptide (intracellular sigma peptide) is being developed as a therapeutic agent for promoting neural regeneration after injury.^25^ Peptides derived from the sequence of the PTPμ wedge domain^14^, ^15^, ^18^ as well as compounds targeting a wedge‐adjacent pocket^26^ have been shown to inhibit the migration of glioma cells in culture. Notably, a compound targeting a wedge‐adjacent pocket was shown to inhibit PTPμ phosphatase activity in vitro.^26^

Like the wedge domains, the D2 domains of RPTPs have controversial functions, but these too have begun to garner interest as potential sites for drug development driven by the recent proliferation of complete crystal structures for RPTP intracellular domains.^27^, ^28^, ^29^, ^30^ Historic studies have indicated that the D2 domain may promote^28^, ^31^, ^32^ or inhibit^33^ the catalytic activity of the D1 domain, may affect substrate specificity,^31^ control protein dimerization^32^, ^33^ or intra‐molecular interactions.^34^ It may also have phosphatase or other enzymatic activities such as a denitrase in some contexts.^35^, ^36^ As of yet, there is no crystal structure encompassing both the D1 and D2 domains of PTPμ to aid drug development; however, in this study, we used a modelling approach, based on the structure of PTPσ, to identify a unique potential drug‐binding pocket that sits at the interface between the D1 and D2 domains. We then used an artificial intelligence neural network (AtomNet® model)^37^ to identify small molecules predicted to interact with this region. The 75 best, chemically diverse, computational hits were tested for the ability to block glioma cell migration and growth as spheres in 3D culture. Active compounds were also tested for their ability to affect PTPμ‐dependent adhesion likely through conformational effects to demonstrate an interaction with the intended target. We identified two high‐priority compounds that inhibited glioma cell migration, sphere formation/growth and PTPμ‐dependent adhesion. We also identified one activator of PTPμ‐dependent adhesion and cancer cell migration. We propose that the high‐priority inhibitors represent unique PTPμ‐targeting agents that can be further developed to treat cancer and that the priority activator may inform how the D2 domain functions to regulate PTPμ.

## 2 METHODS

### 2.1 Cell culture

LN229 (LN‐229), U87 (U87 MG) and Sf9 cells were purchased from the ATCC. The Gli36 (Gli36δ5) glioma cell line^38^ was provided by E. Chiocca and authenticated using IDEXX BioResearch (formerly RADIL: Research Animal Diagnostic Laboratory at the University of Missouri). The human glioma cell lines were cultured in DMEM (high glucose DMEM, Gibco) + 10% (Gli36 and U87) or 5% (LN229) FBS (HyClone) at 37°C and 5% CO_2_. The Sf9 insect cells were cultured in Grace's complete medium (Gibco) + 10% FBS at 27°C.

### 2.2 Scratch‐wound assays

Glioma cells (2.7 × 10^4^ cells per well) were seeded into Incuyte® Imagelock 96‐well plates (Essen BioScience Inc.). Cells were incubated overnight to form monolayers; then, an IncuCyte® 96‐well Woundmaker Tool was used, per the manufacturer's instructions, to generate uniform scratch wounds. Wound closure was observed using the IncuCyte live‐cell imaging system as previously described.^26^

### 2.3 Glioma sphere assays

Ninety‐six‐well U‐bottom plates were coated with 0.75% (wt/vol) PVA as previously described^39^ to create a non‐adherent surface. A Leica CTR6500 microscope fitted with an automated stage was used to capture images on Day 1 and Day 7, and sphere footprint areas were measured using Image J (v1.52a, http://imagej.nih.gov/ij) as previously described.^39^ The effects of the compounds on cell condensation were quantified by calculating the normalized Day 1 footprint areas of the treated wells. Sphere growth was quantified by measuring the change in sphere footprint area (Day 1/Day 7 × 100) and normalizing that to the average size change of the matched DMSO controls. All values are presented as average percentages ± s.e.m.

### 2.4 Helix blue staining

To test for non‐specific toxicity, parental S9 cells (which lack PTPμ) were seeded into 96‐well flat‐bottomed tissue culture plates and treated with compounds (100 μM) or DMSO (1%) for 24 h. To test for glioma cell killing (which could be PTPμ‐dependent), LN229 cells, seeded onto non‐adherent surfaces as described above, were cultured with compounds (100 μM) for 24 h. Sf9 cells and LN229 Day 1 spheres were then stained with 5.5μM helix blue and imaged immediately (Biolegend) on a Leica CTR6500 fluorescence microscope.

### 2.5 Protein tyrosine phosphatase mu‐dependent aggregation assay

Sf9 cells infected with baculovirus coding for human full‐length PTPμ^40^ aggregate following rotation under low shear conditions.^41^ This procedure was adapted to a 48‐well plate format for moderate‐throughput drug screening as described.^19^

## 3 RESULTS

### 3.1 Identification a potential drug‐binding pocket at the interface between the D1 and D2 domains of protein tyrosine phosphatase mu

**FIGURE 1:**
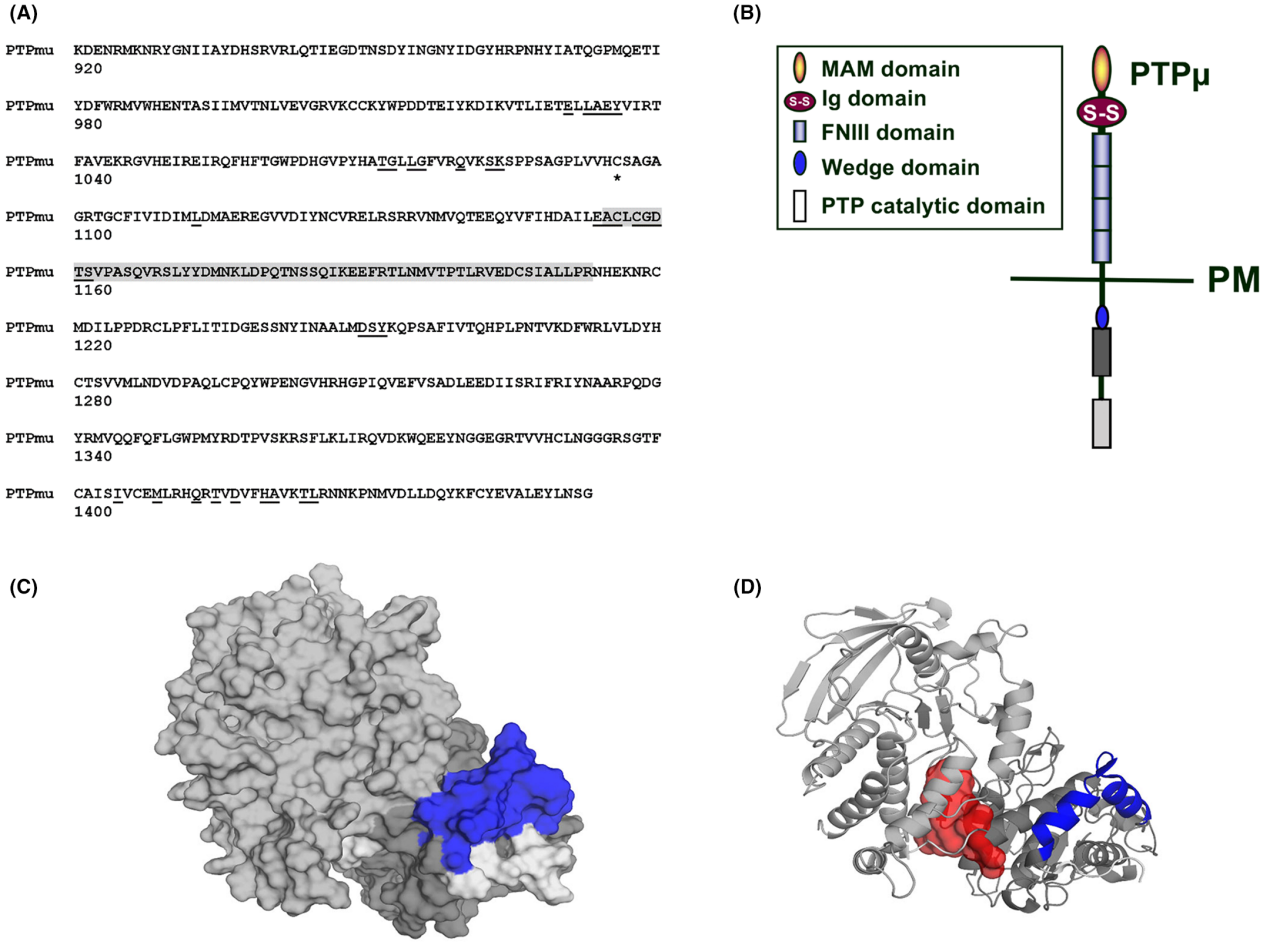
Identification of the D2 wedge binding pocket. (A) The sequence of protein tyrosine phosphatase mu (PTPμ; UniProt P28827‐1). The amino acids that comprise the surface of the binding pocket are underlined. The linker region between the D1 and D2 domains is indicated in grey and the position of the catalytic cysteine in domain 1 is starred. (B). The domain structure of PTPμ with the D1 and D2 phosphatase domains in dark grey and pale grey, respectively, and the known regulatory wedge motif indicated in blue. This colour convention is maintained throughout the figure. (C). A model for the D2 domain of PTPμ relative to the D1 region. The domains are closely associated except for a deep pocket that sits beneath a hook‐shaped projection of the D2 domain. (D). Ribbon structure of the D1 and D2 domains surrounding a space filling model (red) showing the shape of the binding pocket.

To date, all X‐ray structures of RPTPs that contain both D1 and D2 domains show a similar orientation between the two domains, even though the linker region between them may allow for some flexibility in their relative alignment. Prompted by this (and in the absence of a crystal structure that includes the D2 domain of PTPμ), we modelled the D2 domain and parts of the N‐terminal linker domain after the crystal structure for the related PTPσ [PDB ID 2FH7^42^] and combined this with the available PTPμ structure for D1 [PDB ID 1RPM^43^] using ICM (v3.8‐7 Molsoft L.L.C.) as previously described^26^ (Figure 1). Our modelling efforts in a prior study^26^ focused on amino acids surrounding a binding pocket within the D1 domain, while this study focused on surface amino acids comprising an even deeper pocket between the D1 and D2 domains. In both cases, the surrounding amino acids showed strain energies of less than 7 kcal/mol. No attempts were made to resolve every energy strain in regions further away from our binding sites, especially in some of the flexible loop regions, as they were not relevant to the performance of our AtomNet predictions.

Existing crystal structures indicate a close association between the D1 and D2 domains and potential regulatory mechanisms based on dimerization. The crystal structure of the D1 domain of PTPα suggested the protein may exist as an autoinhibited homodimer in which the wedge domain of one molecule occludes the active site of the other.^44^ However, Barr et al.^45^ later showed that this autoinhibited homodimer is incompatible with a complete D1‐D2 structure and suggested a ‘head‐to‐toe’ autoinhibition model for PTPγ, where Asp residues (D1305, D1306) located on the loop connecting the β sheet strands β10 and β11 of the D2 domain, block access to the catalytic site on D1. PTPμ has just one Asp in the corresponding location (D1338, G1339), but in our modelled structure for PTPμ other potential interactions such as D1‐wedge to D2 catalytic site‐homologous region dimers are also generally possible (i.e. they do not lead to steric clashes between the domains). Clearly, a detailed investigation with for example protein–protein docking is necessary to assess the compatibility of the potential interaction sites. We hypothesized that targeting the potential interface between the D1 and D2 domains could lead to the discovery of inhibitors that function by locking the protein in an enzymatically inhibited conformation.

We previously identified a D1 wedge‐adjacent pocket^26^ with drug‐binding/functional activity so we took a similar approach with the D2 domain. We used the ICM pocket finder module (v3.8‐7, Molsoft L.L.C.) to identify a well‐defined pocket at the interface between the D2 and the D1 domains and used the AtomNet® virtual screening platform to identify candidate D2‐binding compounds. Atomwise performed a virtual high‐throughput screen of 4 million compounds from the Mcule small‐molecule library (version v20171018, https://mcule.com/) using their proprietary AI screening AtomNet® platform as described previously.^19^, ^37^, ^46^ Filtering of the 2000 top‐scoring compounds via the removal of compounds with undesired chemical moieties followed by ECFP4 fingerprint‐based Butina clustering (Tanimoto coefficient of 0.4 for similarity cut‐off)^47^ provided a final selection of 75 chemically diverse compounds.

### 3.2 Overview of the functional screen for compounds targeting the protein tyrosine phosphatase mu D2 binding pocket

**FIGURE 2:**
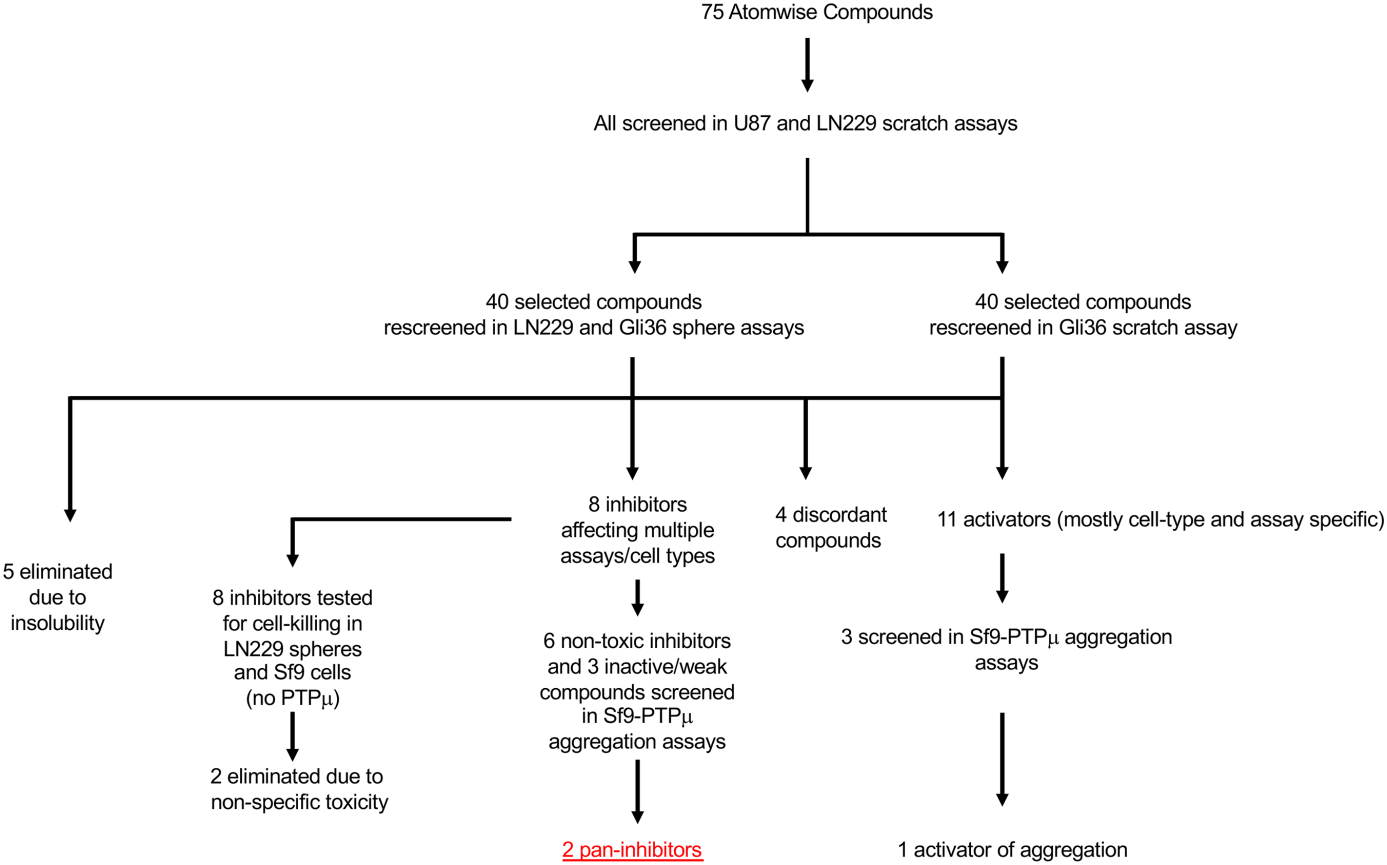
Functional screening approach. Atomwise provided 75 compounds computationally selected for their potential to interact with the D2 binding pocket (D2BP) and two‐blinded DMSO samples. The compounds were screened at 100 μM in scratch wound healing assays using two glioma cell lines (LN229 and U87). A non‐blinded DMSO control was used for normalization purposes in all assays. Active compounds (and some inert controls) were tested for their ability to affect migration of an additional glioma cell line (Gli36) and for their ability to alter LN229 and Gli36 sphere formation and growth. Compounds were eliminated due to insolubility or non‐specific toxicity (based on their ability to kill parental Sf9 cells, which lack PTPμ). Four compounds that were activators in some assays and inhibitors in others were not considered further, while six non‐toxic glioma cell inhibitors and three glioma cell activators were tested in a highly specific assay for PTPμ‐dependent adhesion (Sf9‐PTPμ aggregation). These assays identified two pan‐inhibitors (affecting every cell type and assay) and one activator (that stimulated U87 migration and PTPμ‐dependent aggregation).

**FIGURE 3:**
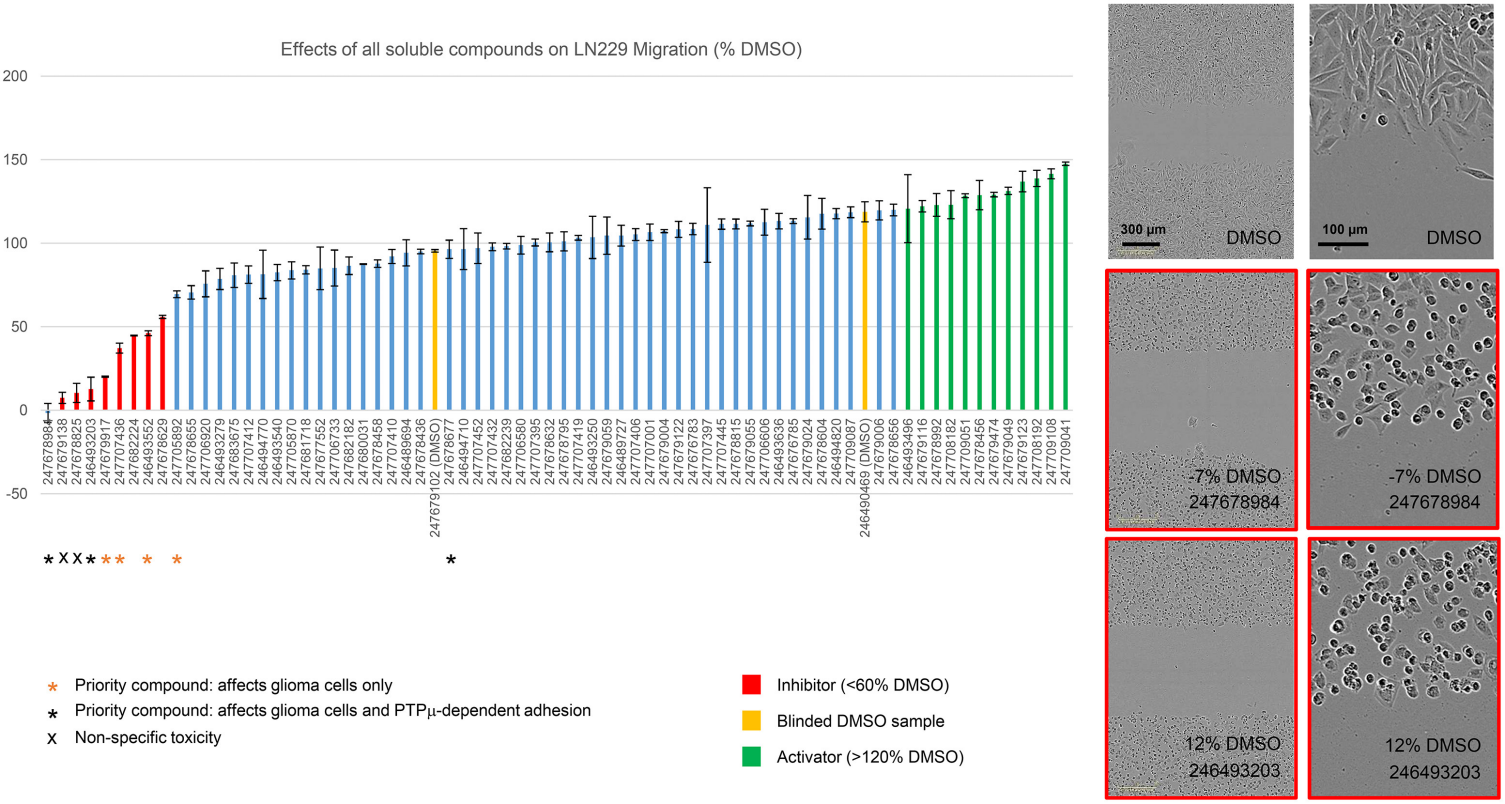
The effects of all soluble D2BP compounds on LN229 scratch wound closure. Start and endpoint scratch‐wound diameters were used to calculate migration distances, which were normalized to the average distance migrated by cells in the non‐blinded DMSO control samples. Data are presented as average percentages ± standard errors of the means (s.e.m.). Compound bar codes are shown on the *x*‐axis. *N* = 2 replicates for most compounds. Some priority hits were screened with *N* = 3–8. Endpoint images of scratch wounds treated with DMSO and two high‐priority inhibitors are shown. The values indicate the relative migration distances of the selected examples.

Figure 2 shows a flowchart of our screening process. Atomwise provided 75 compounds predicted to interact with the D2 binding pocket (D2BP) and two‐blinded DMSO controls. These were screened (at 100 μM) for effects on the migration of two glioma cell lines (LN229, Figure 3 and Figure S1 and U87 Figures S2 and S3) in scratch‐wound assays. These two lines were chosen for the initial screen because they exhibit varying levels of fragment versus full‐length PTPμ. LN229 cells express mostly PTPμ fragments and are invasive in orthotopic tumour models, whereas, U87 cells retain some full‐length PTPμ and are non‐invasive in vivo.^16^, ^48^ A non‐blinded vehicle control (DMSO) was used for normalization purposes in all assays.

**FIGURE 4:**
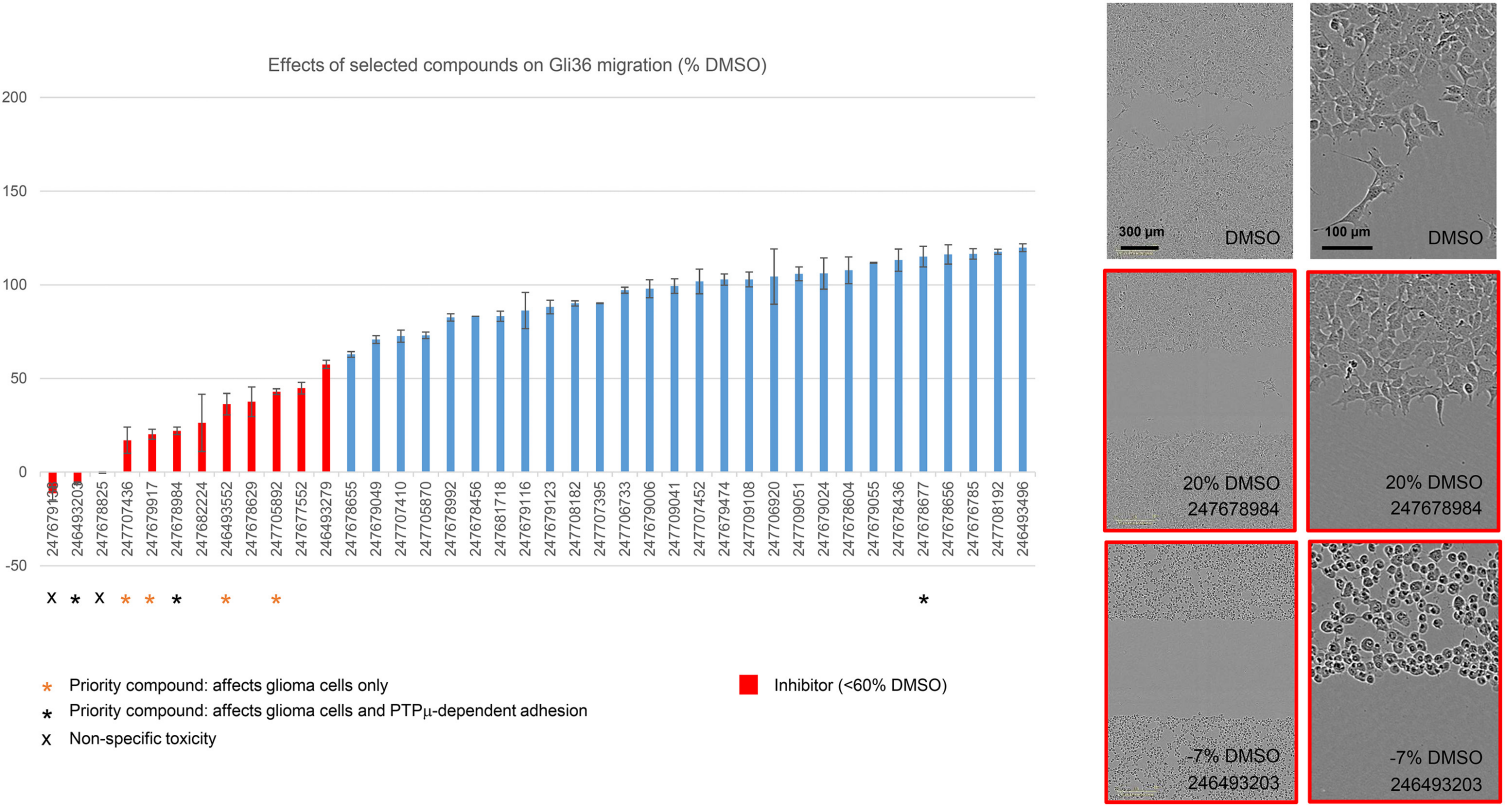
The effects of all selected compounds on Gli36 scratch wound closure. Active compounds from the initial LN229 and/or U87 screens as well as some selected controls were retested at 100 μM for effects on Gli36 migration. The average % movement of the samples relative to the non‐blinded DMSO control is shown ± s.e.m. Compound bar codes are displayed on the *x*‐axis. *N* = 2 replicates for most compounds. Some priority hits were screened with *N* = 4–8 replicates. Endpoint images of scratch wounds treated with DMSO and two high‐priority inhibitors are shown. The values indicate the relative migration distances of the selected examples.

**FIGURE 5:**
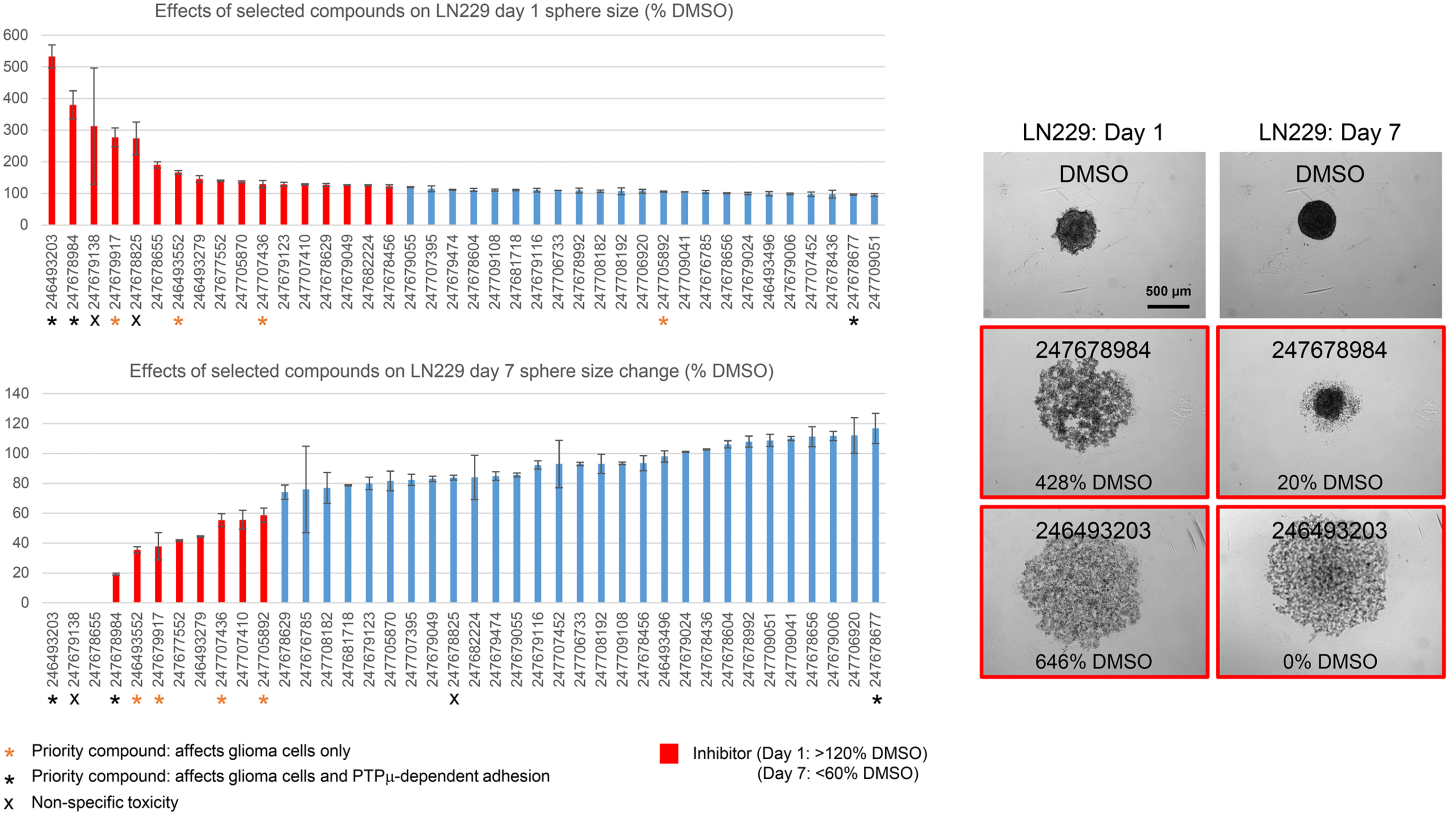
The effects of selected compounds on LN229 sphere formation and growth. Compounds that affected LN229 and/or U87 migration as well as some selected inactive compounds were retested for effects on glioma cell (LN229) sphere formation and growth. LN229 cells cultured on non‐adherent surfaces were treated with compounds (100 μM) or DMSO (1%). The footprint areas of the resulting aggregates were measured on Day 1 and Day 7. Day 1 footprint areas ≥120% that of the controls indicate inhibition of aggregation. Sphere growth was assessed by calculating the change in footprint sizes between Day 1 and Day 7 and normalizing that to the average size change of the DMSO controls. Samples showing ≤60% growth on Day 7 were deemed to be inhibited. Growth could not be calculated for samples that fell apart on Day 1 or during the assay, and this is indicated as 0% growth. Error bars are s.e.m. Most compounds were tested with *n* = 2. Selected compounds were tested with 4–6 replicates. Examples of Day 1 and Day 7 images of samples treated with DMSO and two high‐priority inhibitors are shown. Relative Day 1 footprint areas and Day 7 growth measurements are indicated for each example.

**FIGURE 6:**
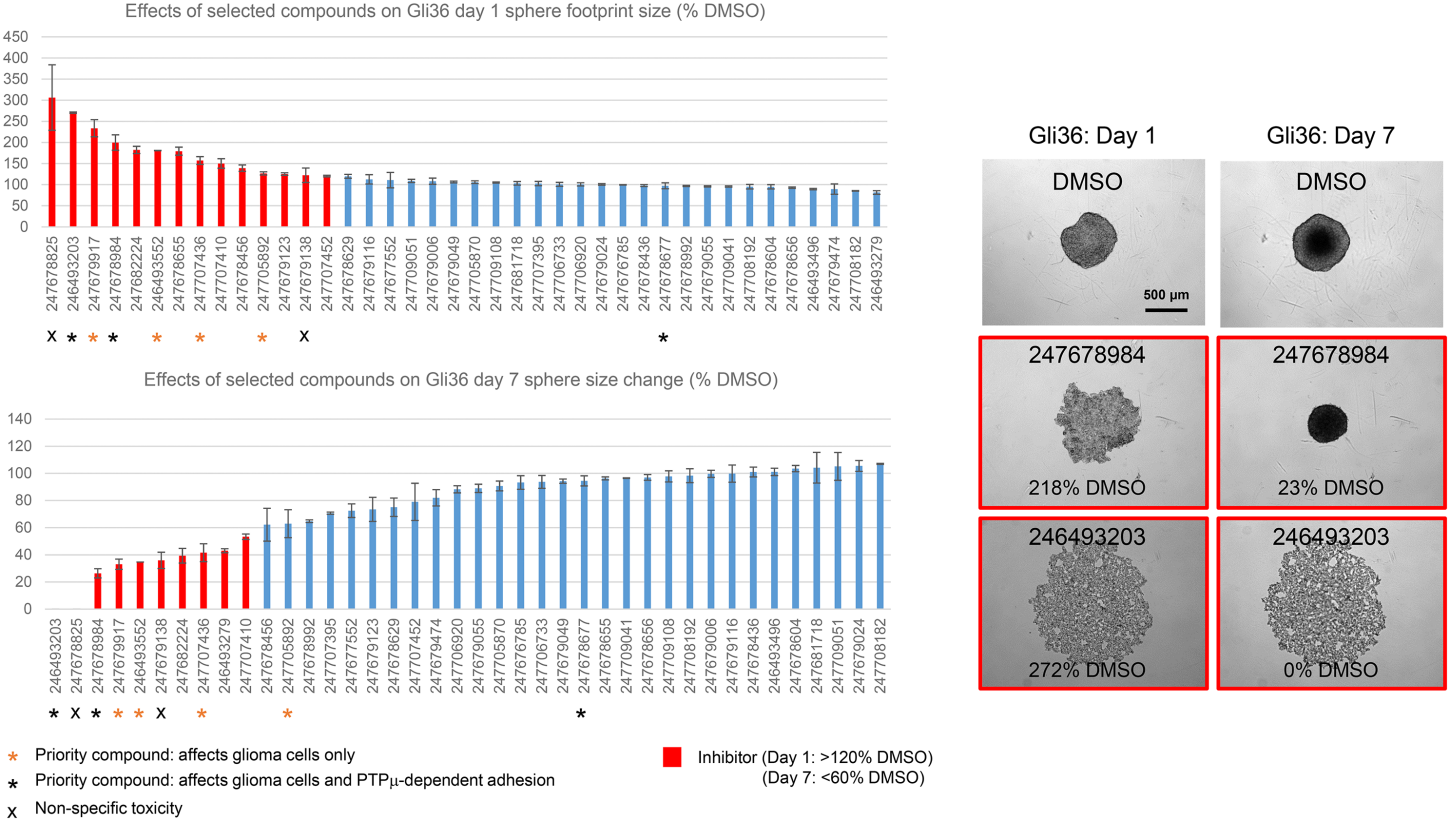
The effects of selected compounds on Gli36 sphere formation and growth. Compounds that affected LN229 and/or U87 migration as well as some inactive control compounds were tested at 100 μM for effects on Gli36 cell sphere formation and growth. Sphere footprint areas were measured on Day 1 and Day 7. The Day 1 data are presented as the average % footprint size relative to the average size of the DMSO controls. The Day 7 data are presented as the average % footprint size change relative to the size change of the DMSO controls. On Day 1, footprint areas ≥120% indicate slowed aggregation, and on Day 7, values ≤indicate reduced growth. The growth of samples that fell apart on Day 1 or during the assay could not be measured, and this is indicated as 0% growth. Error bars are s.e.m. of 2–4 replicates. Representative images of samples treated with DMSO and two high‐priority inhibitors are shown. Relative Day 1 footprint areas and Day 7 growth measurements are given for each example.

**FIGURE 7:**
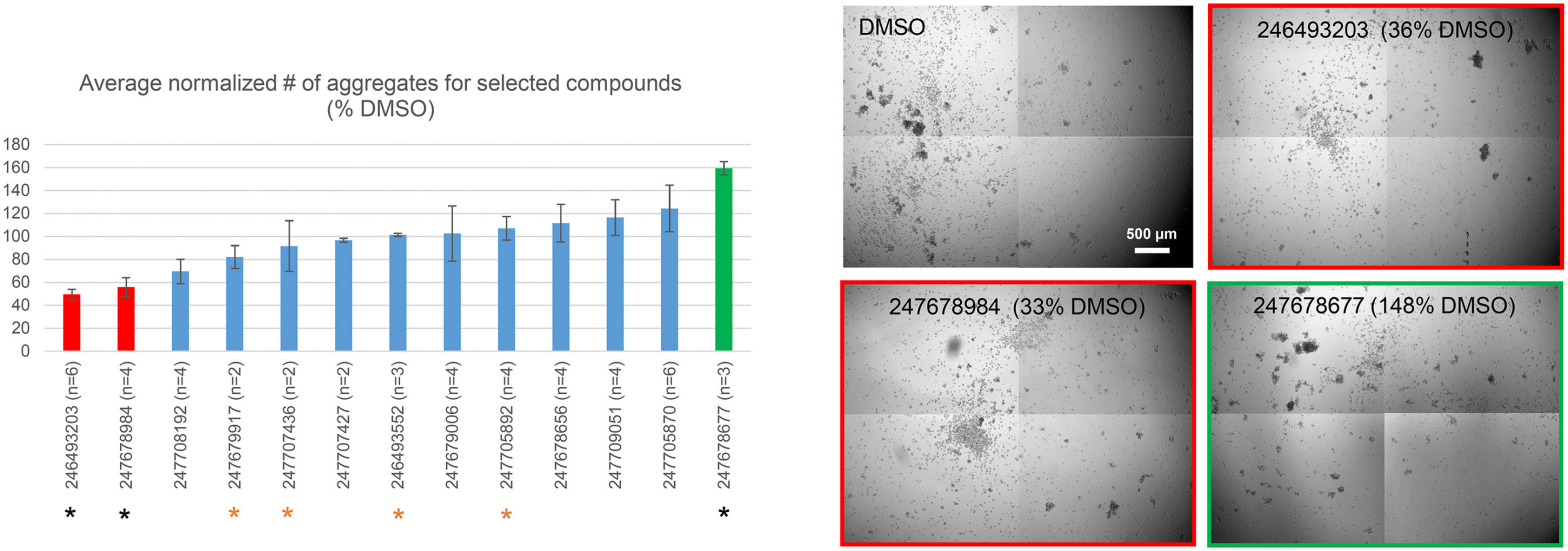
Sf9‐PTPμ aggregation assays. Sf9 cells, which lack RPTPIIb family members, were infected with baculovirus encoding full‐length human PTPμ and cultured for 48 h. Cells were then treated for 20 min with compounds (at 100 μM) or DMSO and induced to aggregate by rotation. The entire surface area of each well was imaged as a 4 × 4 grid, aggregates >4000 μm^2^ were counted and the values normalized to the average number present in the DMSO‐treated controls. Data are presented as percentages ± s.e.m. of the indicated number of replicates. Representative images (central frames) of a DMSO control and samples treated with two priority inhibitors and one priority activator are shown. The relative number of aggregates for each example is shown.

Forty compounds (13 inhibitors of LN229 and/or U87, 14 activators of LN229 and/or U87, one discordant compound [activated U87 and inhibited LN229] and 12 weak or apparently inactive compounds) were further tested for their ability to affect migration of a third glioma cell line (Gli36, Figures 4 and Figure S4) and the aggregation and growth of glioma cells in 3D culture (Figures 5, 6 and Figures S5 and S6). Compounds exhibiting non‐specific toxicity (*n* = 2, Figure S7) or insolubility (*n* = 5, Figure S8; which was typically more apparent in the sphere assay due to the U‐bottom shape of the wells) were eliminated from consideration, and four discordant compounds were not considered further. Finally, a subset of glioma cell inhibitors and activators were tested for their ability to perturb PTPμ‐dependent aggregation of Sf9 cells (Figure 7). Parental Sf9 cells lack PTPμ, as well as other RPTPIIb subtypes and do not aggregate.^19^ However, they can be induced to aggregate by baculoviral‐mediated overexpression of PTPμ, creating a highly specific assay for PTPμ‐dependent adhesion.^41^ We found that two inhibitors that blocked the migration and sphere formation of every tested glioma cell type were also able to block Sf9‐PTPμ aggregation. Additionally, one activator of U87 migration was able to stimulate Sf9 aggregation. Considering the D2 domain is primarily thought to have a regulatory role in controlling intra‐ or inter‐molecular interactions that regulate PTPμ signalling, we had no preformed expectation of identifying D2BP compounds also able to alter aggregation. However, impinging on the D2 domain could change the architecture of the protein in a way that affects presentation of the extracellular segment, and because the Sf9 assay is highly specific for PTPμ, compounds (two inhibitors and one activator) that affect this assay are our highest priority hits and are indicated by black asterisks (Figures 3, 4, 5, 6, 7, Figures S2 and S5). The carbon skeletons of the two high‐priority inhibitors along with docking models and estimated Gibb's free energies of binding are presented in Figure S9. The four compounds that inhibited glioma cells but not PTPμ‐mediated aggregation may still have therapeutic potential and are marked by orange asterisks. They may alter PTPμ's signalling function without altering adhesion or act via another mechanism.

### 3.3 Glioma cell scratch assays

Cancer cell motility was evaluated by scratch assays where cells migrate into a wound and close it creating a monolayer. The effects of all soluble D2BP compounds on LN229 scratch wound closure are shown in Figure 3. In this initial screen, nine strong LN229 inhibitors were identified (red bars), and of these, six were eventually prioritized for being effective in multiple cell types and assays (Figure 2). We also identified 12 activators (≥120% of DMSO closure rate) of LN229 wound closure, only one of which (246493496) was shared with U87 cells (Figure S2). In general, the U87 cells appeared less ‘activatable’, with only four compounds stimulating their migration by ~1.2–1.4×, one of which (247678677) was later shown to affect PTPμ‐dependent adhesion. Thus, 247678677 is indicated as a priority compound in Figure 2 despite not affecting LN229 migration. It is possible that this compound was a U87‐specific activator because these cells express more full‐length PTPμ than LN229 cells.

Figure 3 shows examples of the morphology of LN229 cells that were treated with DMSO (1%) or two high‐priority inhibitors (100 μM). Within DMSO‐treated monolayers, the LN229 cells were generally spindle shaped, whereas cells at the wound edge were flattened and showed lamellipodial ruffles consistent with being migratory. The two high‐priority inhibitors caused LN229 cells to round up. 246493203 caused similar morphological changes in U87 cells, but 247678984 had a more modest effect on this cell type, and there was little difference in morphology between U87 cells treated with this compound and control cells (Figure S2).

The morphological effects of the low‐priority inhibitors on LN229 and U87 cells are shown in Figures S1 and S3, respectively, and were generally quite subtle. LN229 cells within monolayers treated with 247679917, 247707436 and 247705892 appeared more spread than spindle shaped, creating a cobblestone appearance, and cells at the edges of the scratches exhibited few ruffles consistent with reduced motility. 246493552 reduced the appearance of ruffles at the scratch edge but did not affect the overall appearance of the monolayer. Some rounding of U87 cells was apparent with 247679917 and 246493552, but 247707436, despite being strongly inhibitory, did not affect the morphology of this cell type. The U87‐specific priority activator did not produce obvious morphological changes.

A selection of 40 compounds (those shown to inhibit or activate the migration of one or both cell types and some inert control compounds) were tested for effects on Gli36 cells (Figure 4). This line expresses mostly PTPμ fragments and migrates rapidly in vitro (requiring scratch‐wound endpoint images to be taken at 8 h versus the typical 12‐h time frame for the U87 and LN229 experiments). Despite this, the majority of rescreened inhibitors were effective on this cell line; however, no Gli36 activators were identified. Considering the rapid migration of Gli36 cells, they may not be particularly sensitive to further stimulation.

Representative endpoint images of Gli36 scratch wounds treated with DMSO or the two high‐priority inhibitors are shown in Figure 4. Gli36 cells in control monolayers have a cobblestone appearance, while those at the scratch edge appear to extend process and move into the scratch as linked chains of cells. The strong priority inhibitor 246493203 caused cell rounding while the more moderate inhibitor 247678984 reduced the appearance of cell chains moving into the scratch. The effects of the low‐priority inhibitors are shown in Figure S4. Of these, only 247707436 produced a marked change in cell morphology. It completely blocked the formation of processes and cell chains at the edge of the scratch.

### 3.4 Glioma cell sphere assays

Glioma cells cultured on non‐adherent surfaces will self‐adhere and grow in culture as a compact sphere that mimics some aspects of the 3D tumour microenvironment.^39^ Agents able to affect the growth and survival of 3D cultures may be more potent in vivo anti‐tumour agents than those just able to affect cells in 2D culture.^49^ Also, culture systems that favour cell–cell over cell‐substrate adhesion are likely better models for understanding the role of cell‐cell adhesion molecules in cancer biology. Thus, the effects of 40 compounds (13 shown to inhibit the migration of at least one cell type, 14 shown to stimulate migration of at least one cell type, one discordant compound [activated U87 and inhibited LN229] and 12 weak or apparently inactive compounds) were tested for their ability to affect LN229 sphere formation and growth (Figure 5). To quantify sphere formation, the footprint areas occupied by the cells were measured on Day 1, and the values normalized to the average footprint area of the DMSO controls. Larger footprint sizes (>120%) on Day 1 indicate inhibition, i.e. the cells have failed to compact. Sphere growth was quantified by measuring the percent changes in sphere footprint areas between Day 1 and Day 7 and normalizing those to the average growth of the DMSO controls. On Day 7, spheres exhibiting a size change ≤60% of controls were considered inhibited. Samples that fell apart on Day 1 or during the culture period are displayed as having 0% growth.

Representative images of LN229 spheres cultured in the presence of DMSO or the two high‐priority inhibitors are shown in Figure 5. The LN229 cells treated with DMSO formed a loose aggregate on Day 1, and by Day 7, this aggregate had formed a compact sphere. In contrast, the cells treated with a strong inhibitor (246493203) failed to compact and remained as single cells at the bottom of the well. Samples treated with the more moderate inhibitor (247678984) formed scattered clumps on Day 1 that eventually compacted into a small aggregate by Day 7. All six prioritized inhibitors were titrated to estimate a minimal dose (25, 50 or 100 μM) able to affect sphere formation and/or growth (Figure S5). The strong inhibitor 246493203 dramatically disrupted sphere formation at 100 μM and 50 μM. At 25 μM, sphere formation was modestly perturbed, but the resulting aggregates grew poorly in culture. The other high‐priority inhibitor (247678984) was most effective at 100 μM where it slowed both sphere condensation and growth. At lower doses, that compound still had a modest effect on sphere formation, but the samples recovered and grew normally. The low‐priority inhibitors (orange asterisks) were only effective at 100 μM, and representative images of samples treated with these compounds are shown in Figure S6. Three of these compounds (247707436, 246493552 and 247705892) produced very similar effects: sphere formation was modestly slowed, and the resulting aggregates grew poorly in culture. 247679917 caused a distinct effect. Cells treated with this compound aggregated poorly but eventually condensed into a clump with irregular loose‐appearing margins.

The effects of the priority inhibitors on Gli36 sphere formation and growth were generally similar (i.e. treatment with the compounds slowed condensation and/or growth; Figure 6 and Figure S6). 247679917 was again distinct. Like LN229 cells, Gli36 cells responded to this compound by forming loose‐appearing clumps with irregular borders.

### 3.5 Protein tyrosine phosphatase mu‐dependent aggregation assays

The glioma cell assays described above involve hours (scratch assays) to many days (sphere assays) of treatment and a very high dose of compounds, raising the possibility of off‐target effects. To maximize our chances of identifying PTPμ‐targeting compounds, we screened selected hits in a short‐term assay for PTPμ‐dependent adhesion. RPTPIIb family members are not expressed in parental Sf9 cells,^19^ and these cells do not aggregate. However, they can be induced to rapidly aggregate in response to baculoviral‐driven expression of PTPμ, providing a highly specific measure of PTPμ function.^41^ It is unclear how the D2 domain might impact adhesion, but it could participate in interactions that affect PTPμ oligomerization or association with downstream targets, thereby altering the PTPμ‐adhesive complex. To test if our D2BP‐targeted compounds affect adhesion, PTPμ‐expressing Sf9 cells were treated with selected compounds (100 μM) for 20 min then induced to aggregate by rotation. Aggregates above an arbitrary threshold size (4000 μm^2^) were then counted and normalized to the number present in the DMSO‐treated controls. Endpoint images of samples treated with DMSO, two priority inhibitors and one priority activator are shown in Figure 7. The DMSO‐treated sample exhibits many variable‐sized aggregates, whereas, the samples treated with the high‐priority inhibitors (247678984 and 246493203) have fewer/smaller aggregates. In contrast, samples treated with the U87 activator 247678677 (Figure S2) exhibit an increase in the number of intermediate‐sized aggregates.

## 4 DISCUSSION

Through computational modelling, we have identified a pocket at the interface between the D1 and D2 domains of PTPμ that can be exploited for drug development. Compounds predicted to interact with this region were identified through an AI‐based computational algorithm and tested for their ability to affect tumour cell migration, growth in 3D culture and PTPμ‐dependent adhesion. We identified two compounds that inhibited multiple glioma cell types and reduced PTPμ‐dependent adhesion, the strongest of which (246493203) was effective on glioma cell spheres down to 25 μM. We also identified four low‐priority inhibitors that inhibited glioma cell migration and sphere formation/growth without affecting PTPμ‐aggregation and one activator of PTPμ‐dependent aggregation that also stimulated U87 migration. We propose that the high‐priority inhibitors may serve as a starting point for developing PTPμ‐specific therapeutic agents. The low‐priority inhibitors may affect PTPμ‐signalling without altering PTPμ‐dependent adhesion or act via other mechanisms. They may still be therapeutically useful considering their ability to inhibit glioma cells in culture, but they were only active at very high concentrations, meaning they would require considerable refinement. The activator is also interesting, not from a therapeutic standpoint for cancer cells, but because it might inform how the D2 domain functions to regulate PTPμ. Notably, the results of this screen were very similar to those of a prior screen for compounds targeting a D1 wedge‐adjacent binding pocket.^26^ In each screen, we recovered inhibitors able to affect both glioma cell migration and PTPμ‐dependent adhesion and activators with the converse effect. These correlations led us to hypothesize that cancer cells may actually receive a growth/motility benefit from increased PTPμ‐mediated adhesion. This is in contrast to the tumour suppressive function proposed for PTPμ and may indicate that glioma cells have subverted the normal adhesive role of full‐length PTPμ (in contact inhibition)^2^ into adhesive signals, possibly mediated by shed fragments, that promote cancer progression.

We do acknowledge that cell‐based assays are complex, particularly in the context of cell lines expressing different ratios of full‐length PTPμ and its fragments. However, biochemical assays, including measurements of phosphatase activity or direct binding, cannot recapitulate the range of possible activities mediated by PTPμ. In particular, a myriad of functions have been proposed for the D2 domain of RPTPs, and reductionist assays focusing on one model might miss active compounds that perturb different activities. Most studies of the D2 domain have focused on its regulatory function with respect to the D1 domain, with data supporting both stimulatory and inhibitory activities. The crystal structure of the LAR D1 and D2 domains revealed that they are closely associated,^28^ so much so that the N‐terminal residues of the D2 domain form the backside of the D1 catalytic site and are necessary for phosphatase activity. Consistent with this, a LAR construct lacking the D2 domain was not catalytically active on the preferred MBP substrate, but activity was restored by adding back the first 102 amino acids of the D2 domain.^31^ Streuli et al.^31^ also performed a careful analysis of a series of deletion constructs of the cytoplasmic domain of LCA (CD45) and LAR and demonstrated that internal deletions within the D2 domain can affect substrate specificity, at least in in vitro phosphatase assays. Ng et al.^50^ also showed that mutations in the D2 domain of CD45 can inhibit its phosphatase activity, suggesting the D2 domain is necessary for activation.

The D2 domain may activate or inhibit catalytic activity by regulating RPTP dimerization. A bacterially expressed construct comprising the entire cytoplasmic tail of CD45 was more catalytically active than a construct containing just the D1 domain. The D1‐D2 construct was demonstrated to be monomeric, whereas the D1 construct formed a dimer, leading to a model in which the D2 domain potentiates activity by blocking dimerization.^32^ In contrast, FRET was used to show that PTPα can dimerize in living cells,^51^ and that the D2 domain (along with many other domains) may facilitate this interaction at least in the context of transiently overexpressed proteins. Jiang et al.^33^ proposed that dimerization inhibits PTPα's phosphatase activity indicating the D2 domain may have an inhibitory, rather than an activating, function. The D2 domain might also convey inhibition by mediating trans interactions. The D1 domain of PTPσ was shown to be inhibited by interactions with the D2 domain of PTPδ.^52^ In the context of PTPμ, the D2 domain has been proposed to participate in intramolecular interactions that control activity. Both the D1 and D2 domains of PTPμ were shown, by yeast 2 hybrid, to interact with the juxtamembrane portion of the protein.^34^ The D2‐juxtamembrane interaction was proposed to be inhibitory and predicted to act by blocking necessary contact between the D1 and juxtamembrane regions. Unfortunately, there is currently no crystal structure for the full‐length cytoplasmic region of PTPμ to support these possible interactions. However, in our model of the possible structure (as informed by that of PTPσ) various interactions between the D1 and D2 domains are sterically feasible and likely perturbable by agents targeting the interface between these two domains.

Despite the intense focus on the various trans and cis interactions proposed for the D2 domain, its actual role remains uncertain. Most of the proposed interactions have not been confirmed in vivo in the context of endogenous expression levels, and most were not assessed in the presence of the transmembrane and extracellular regions of the proteins. It should also be noted that despite in vitro evidence indicating that the D2 domain is phosphatase‐inactive, mutational analysis suggests that it could be poised to function and might have activity in the correct cellular context or with the correct substrates.^28^ Of note, the D2 domain of PTPα does have slight phosphatase activity in vitro.^35^ It could also be true that the D2 domain may be configured to remove other tyrosine modifications such as denitration, as proposed for PTPRT.^36^ Finally, RPTPs have functions that do not require phosphatase activity. A phosphatase‐inactive form of PTPμ was shown to be able to restore E‐cadherin‐mediated cell–cell adhesion in prostate cancer cells. This activity required the intracellular domain and was likely mediated through PTPμ interacting with RACK1.^53^ Our compounds could, conceivably, impinge on any number of these inter‐ or intra‐molecular binding activities, producing changes via phosphatase‐ or non‐phosphatase‐dependent processes. Considering this, it is best to cast a wide net for screening for active compounds, which we have attempted by performing cell‐based assays under conditions that recapitulate the PTPμ fragments seen in cancer cells.

Additional studies are necessary to confirm the mode of action of our high‐priority hits, including addressing whether these compounds alter the signalling activity of PTPμ. Also, direct binding assays will be performed to confirm the interaction of compounds with the D2 binding pocket and, considering the structural conservation of the D2 domains, whether these compounds are specific to PTPμ. Notably, Perron et al.^54^ took a similar in silico screening approach targeting a pocket at the interface between the D1 and D2 domains of CD45 and identified a highly specific allosteric inhibitor. The region described by them appears to be in the same general area of the protein as the one targeted by us. Given the low conservation of residues in this region between PTPμ and other RPTPs, we may have identified agents that are similarly specific for PTPμ. Future directions should also include the structure–activity relationship studies necessary to improve the activity of our priority hits. Finally, the compounds will need to be tested in tumour models to determine their therapeutic potential.

## 5 CONCLUSIONS

We have identified small molecules predicted to interact with a unique regulatory site within the D2 domain of PTPμ that inhibit PTPμ‐dependent adhesion, glioma cell migration, sphere formation and growth in 3D culture. These compounds may serve as the basis for developing new treatments for cancers where the function of PTPμ is dysregulated, including glioblastoma^18^ and gynaecological cancers.^55^

## AUTHOR CONTRIBUTIONS

**Kathleen Molyneaux:** Formal analysis (lead); investigation (lead); methodology (equal); writing – original draft (lead); writing – review and editing (equal). **Christian Laggner:** Methodology (equal); software (lead); writing – review and editing (equal). **Susann M. Brady‐Kalnay:** Conceptualization (lead); funding acquisition (lead); project administration (lead); supervision (lead); writing – review and editing (equal).

## FUNDING INFORMATION

This study was supported with an AIMS Award (A17‐020 to Susann M. Brady‐Kalnay) from Atomwise, covering the supply and delivery of the compounds.

## CONFLICT OF INTEREST STATEMENT

Susann M. Brady‐Kalnay and Kathleen Molyneaux declare no competing interests. Christian Laggner is an employee of Atomwise.

## Supporting information


Data S1.

Figure S1

Figure S2.

Figure S3

Figure S4.

Figure S5

Figure S6

Figure S7

Figure S8

Figure S9

## Data Availability

All data generated or analysed during this study are included in this published article (and its supplementary information files). Materials are available upon request.
