# Supplementary material for: A novel binding pocket in the D2 domain of protein tyrosine phosphatase mu (PTPmu) guides AI screen to identify small molecules that modulate tumour cell adhesion, growth and migration
Source: J Cell Mol Med. 2023 Oct 20;27(22):3553–64. doi: 10.1111/jcmm.17973 (PMC10660673; doi:10.1111/jcmm.17973)
Supplement: Supplementary file 1 — Data S1. [file JCMM-27-3553-s004.docx]

**Supporting Information**

**S1 Fig. LN229 scratch wounds treated with DMSO or selected lower priority inhibitors.** The relative migration distances (% DMSO control) are shown for each example.

**S2 Fig. The effects of all soluble D2BP compounds on U87 scratch wound closure.** Migration was quantified from start and end-point scratch wound widths and is presented as the normalized % migration + s.e.m. relative to the DMSO controls. Compound bar codes are shown on the x-axis. All compounds were screened with an n of 2. Examples of samples treated with DMSO or two high priority inhibitors are shown.

**S3 Fig. Endpoint images of U87 scratch wounds treated with DMSO or selected lower priority compounds.** The distance moved relative to controls for each example is indicated.

**S4 Fig. Example endpoint images of Gli36 scratch wounds treated with lower priority inhibitors.** The distance moved relative to controls is indicated for each example.

**S5 Fig. Titration of selected compounds on LN229 sphere formation and growth.** LN229 cells plated on non-adherent surfaces were treated with the indicated doses of compounds. The initial hit at 100 μM and the follow-up titration (at 100 μM, 50 μM, and 25 μM) are shown. Sphere footprint areas were measured on day 1 and day 7. Day 1 data is presented as the normalized sphere footprint area (% DMSO) +. s.e.m. of two replicates. Day 7 data is presented as the normalized size change (% DMSO) in sphere footprint areas + s.e.m. of two replicates. For samples that fell apart on day 1 or during the assay, no growth could be measured, and this is indicated as 0%. Example images of samples treated with two priority inhibitors are presented and their relative day 1 footprint areas and day 7 growth are indicated.

**S6 Fig. Examples of LN229 and Gli36 spheres cultured in the presence of DMSO or the indicated lower priority inhibitors (100 μM).** The relative day 1 footprint area and day 7 size change for each compound are indicated.

**S7 Fig. Testing for compounds that affect cell viability.** LN229 cells cultured on non-adherent surfaces or parental Sf9 cells (which lack PTPµ) were treated for 24 h with the indicated compounds (100 µM). Samples were then stained with Helix Blue to detect dying cells. Two compounds showed non-specific toxicity to Sf9 cells and were eliminated from the screen. The six prioritized inhibitors did not induce cell death in glioma cells or in Sf9 cells. Examples of two untreated LN229 spheres are shown because there is variability in the level of Helix Blue staining among control samples.

**S8 Fig. Examples of insoluble compounds.** Five compounds were eliminated from the screen due to insolubility at 100 μM. This effect was more obvious in the sphere assay where black material/dust accumulated within and around spheres at the bottom of the well. 247679046 produced visible puncta in the scratch assay. In the sphere assay, these puncta appeared to nucleate the formation of multiple clumps. 247706561 precipitated in scratch assays and was not tested on spheres.

**S9 Fig. Carbon skeletons and docking poses for the highest priority inhibitors.** A. The carbon skeleton (CS) of 247678984 and the best docking poses from MCule 1-Click docking (<https://mcule.com/apps/1-click-docking/>) using Thr1071 as the binding center. The predicted Gibb’s free energy of binding is -9.3 kcal/mol. B. The carbon skeleton of 246493203 and its best docking pose. The predicted Gibb’s free energy of binding is -9.4 kcal/mol.
