# Supplementary figures and images for: A novel binding pocket in the D2 domain of protein tyrosine phosphatase mu (PTPmu) guides AI screen to identify small molecules that modulate tumour cell adhesion, growth and migration
Source: J Cell Mol Med. 2023 Oct 20;27(22):3553–64. doi: 10.1111/jcmm.17973 (PMC10660673; doi:10.1111/jcmm.17973)

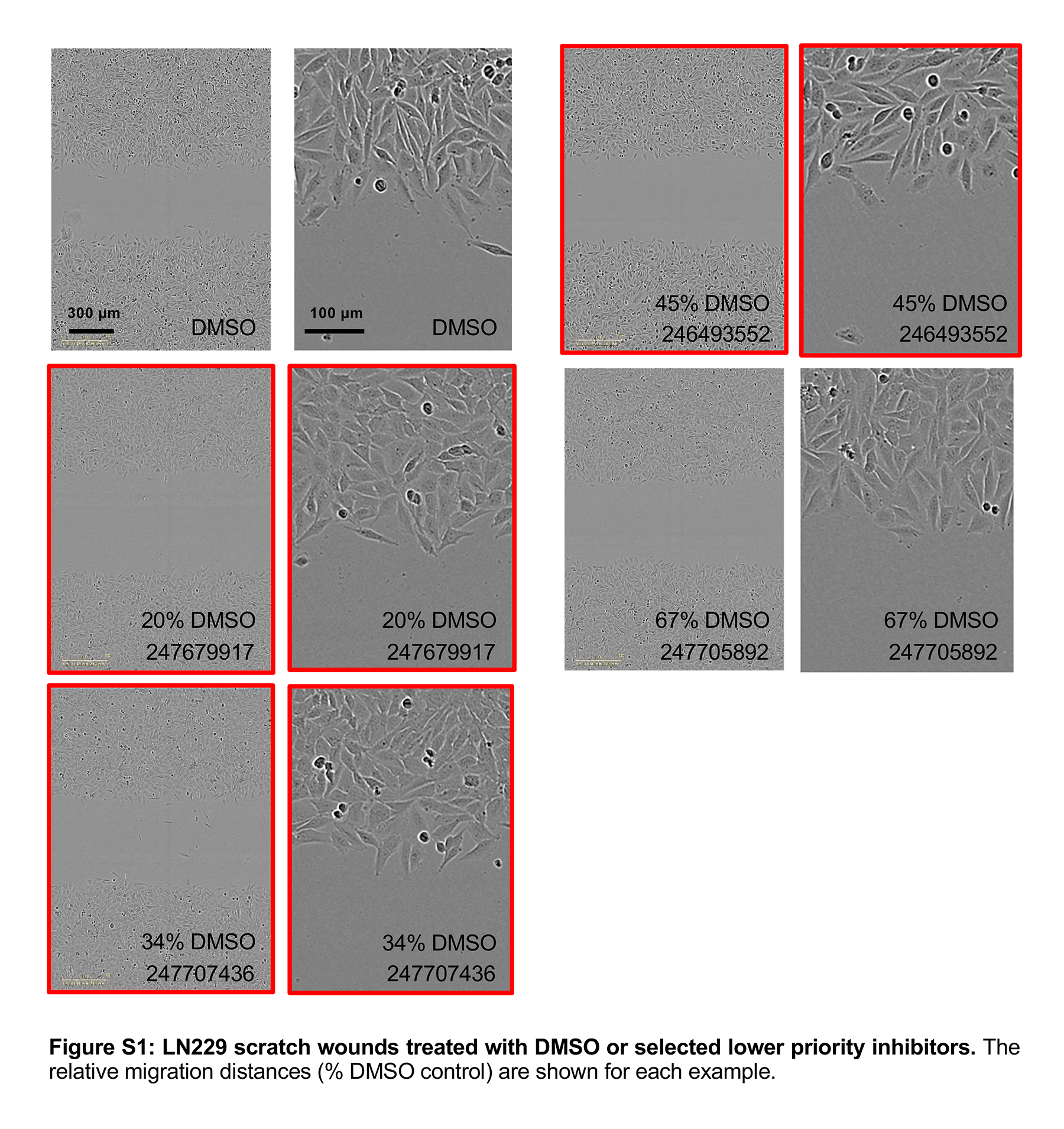

Supplement: Supplementary file 2 — Figure S1 [file JCMM-27-3553-s002.tif]

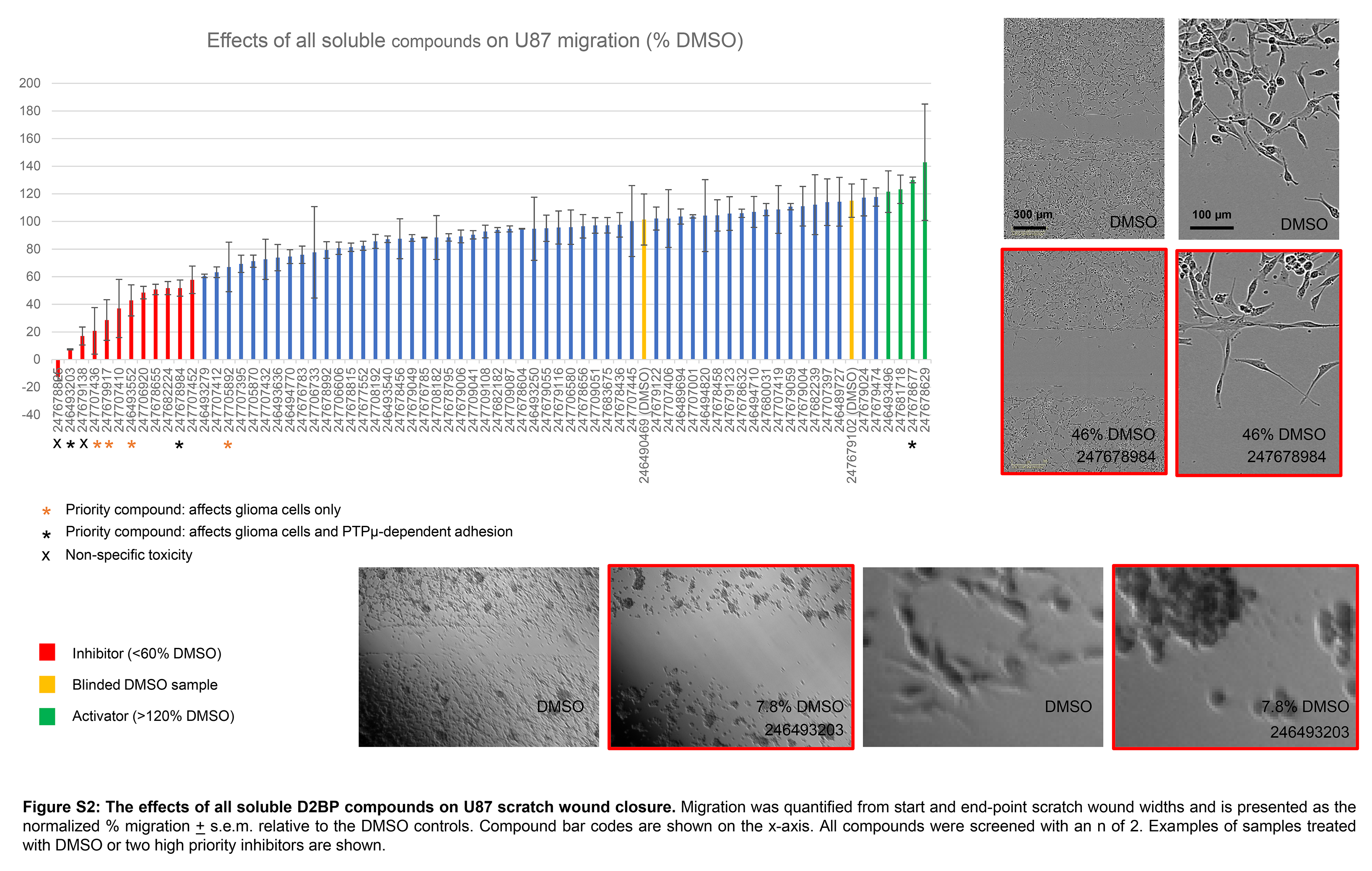

Supplement: Supplementary file 3 — Figure S2. [file JCMM-27-3553-s008.tif]

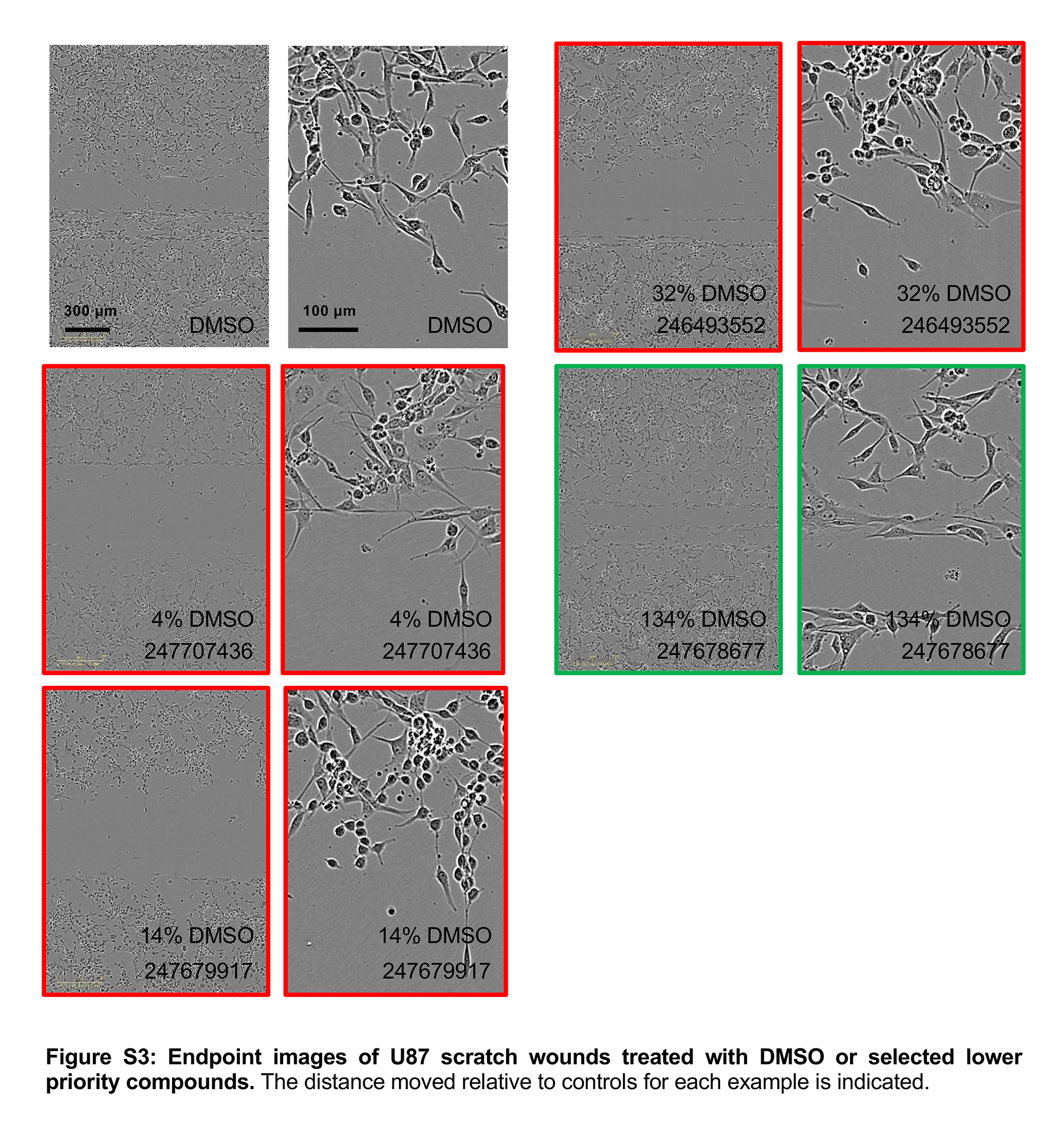

Supplement: Supplementary file 4 — Figure S3 [file JCMM-27-3553-s009.tif]

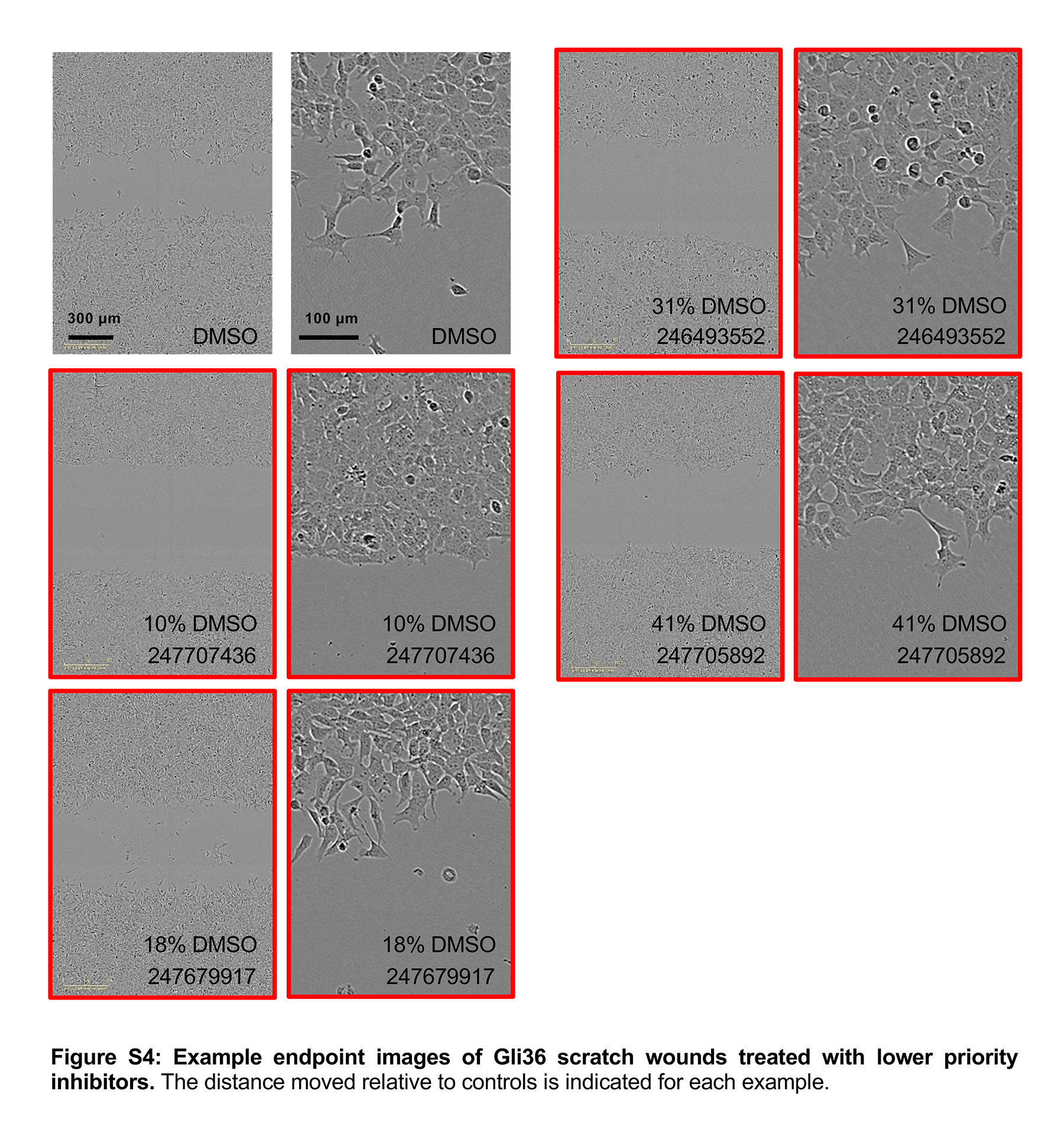

Supplement: Supplementary file 5 — Figure S4. [file JCMM-27-3553-s010.tif]

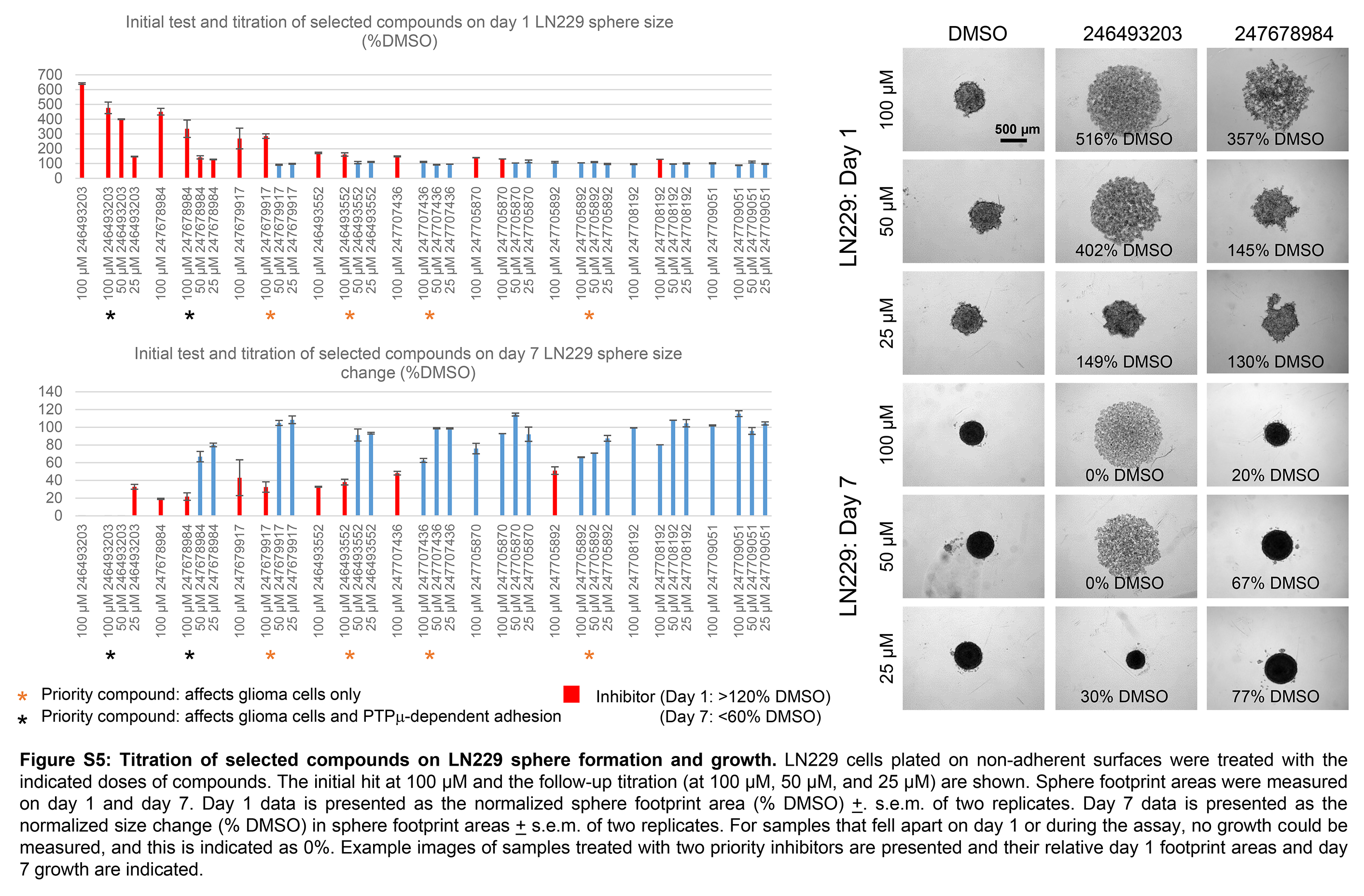

Supplement: Supplementary file 6 — Figure S5 [file JCMM-27-3553-s005.tif]

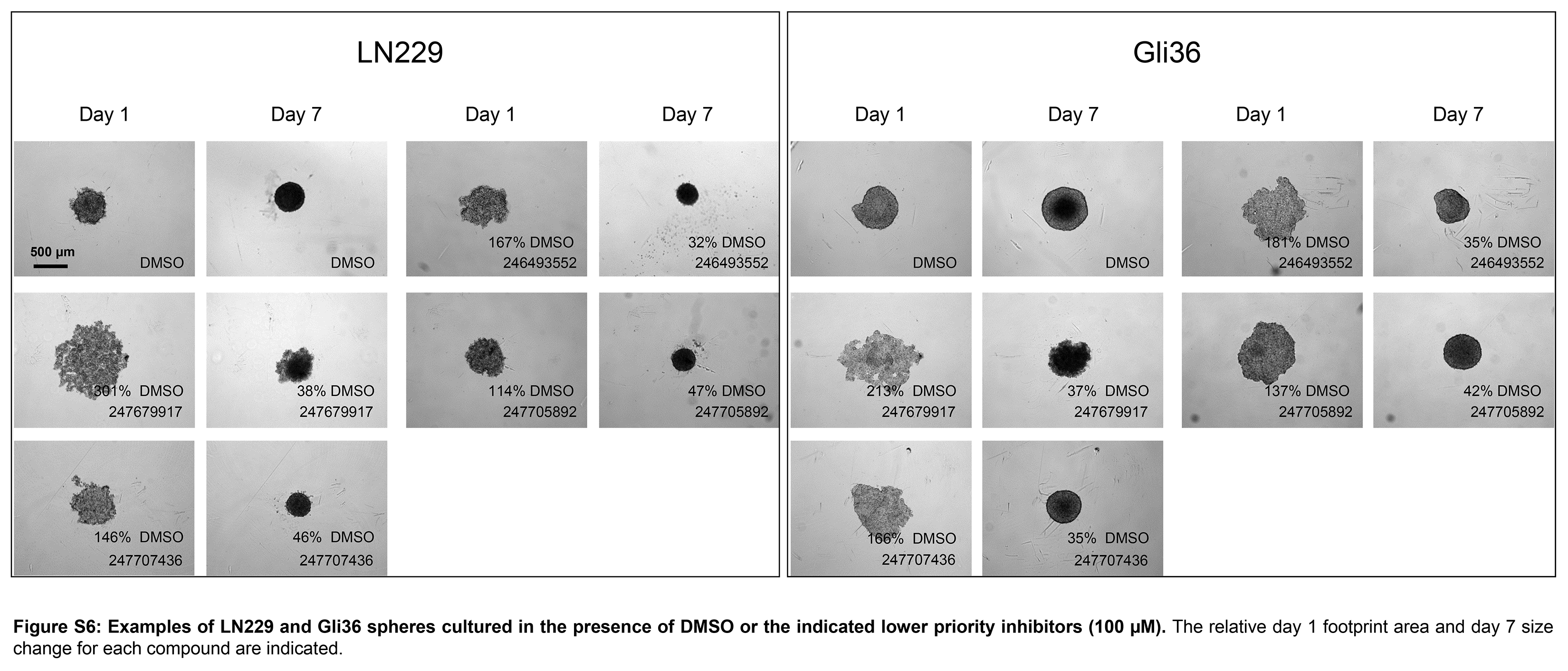

Supplement: Supplementary file 7 — Figure S6 [file JCMM-27-3553-s006.tif]

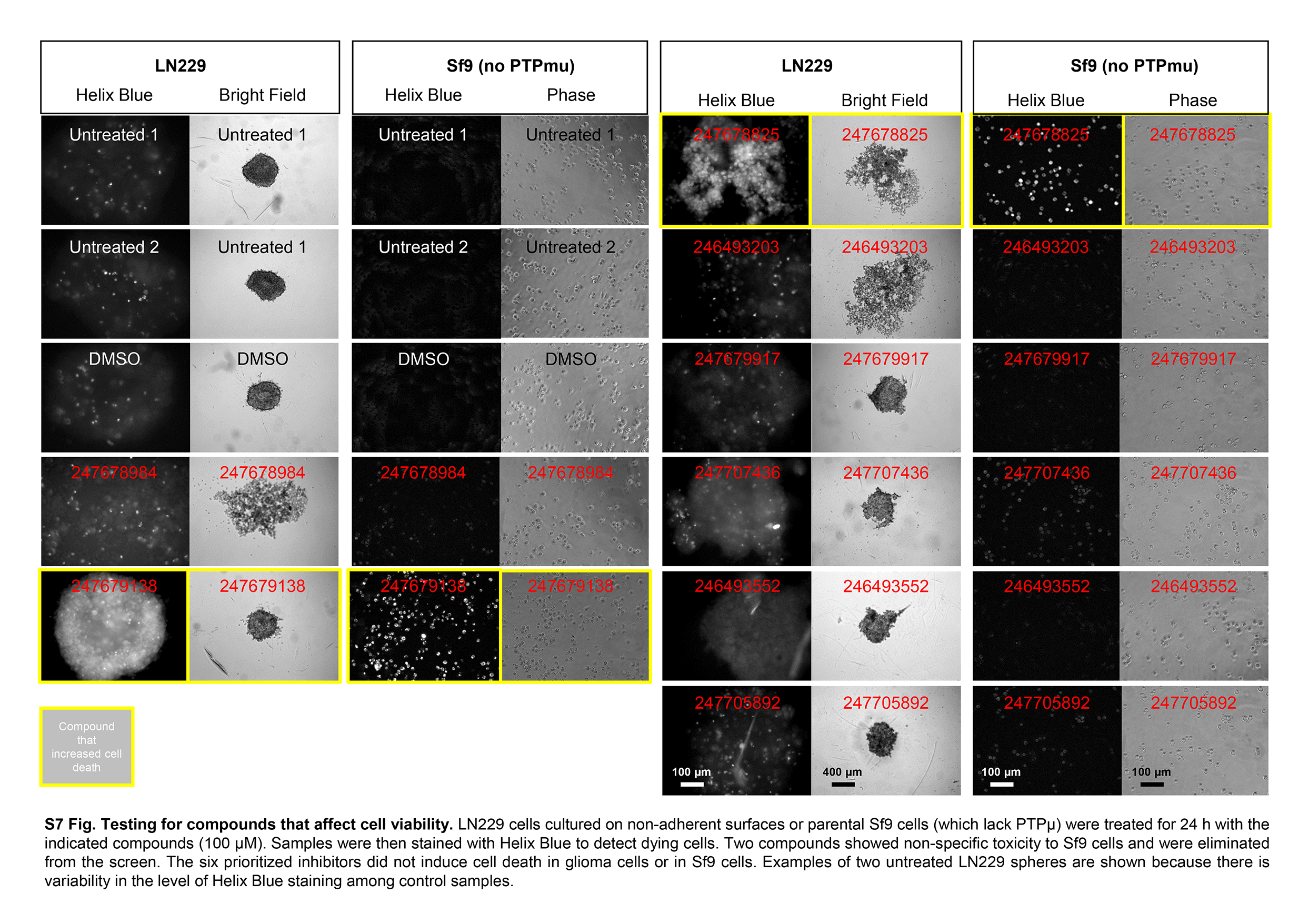

Supplement: Supplementary file 8 — Figure S7 [file JCMM-27-3553-s001.tif]

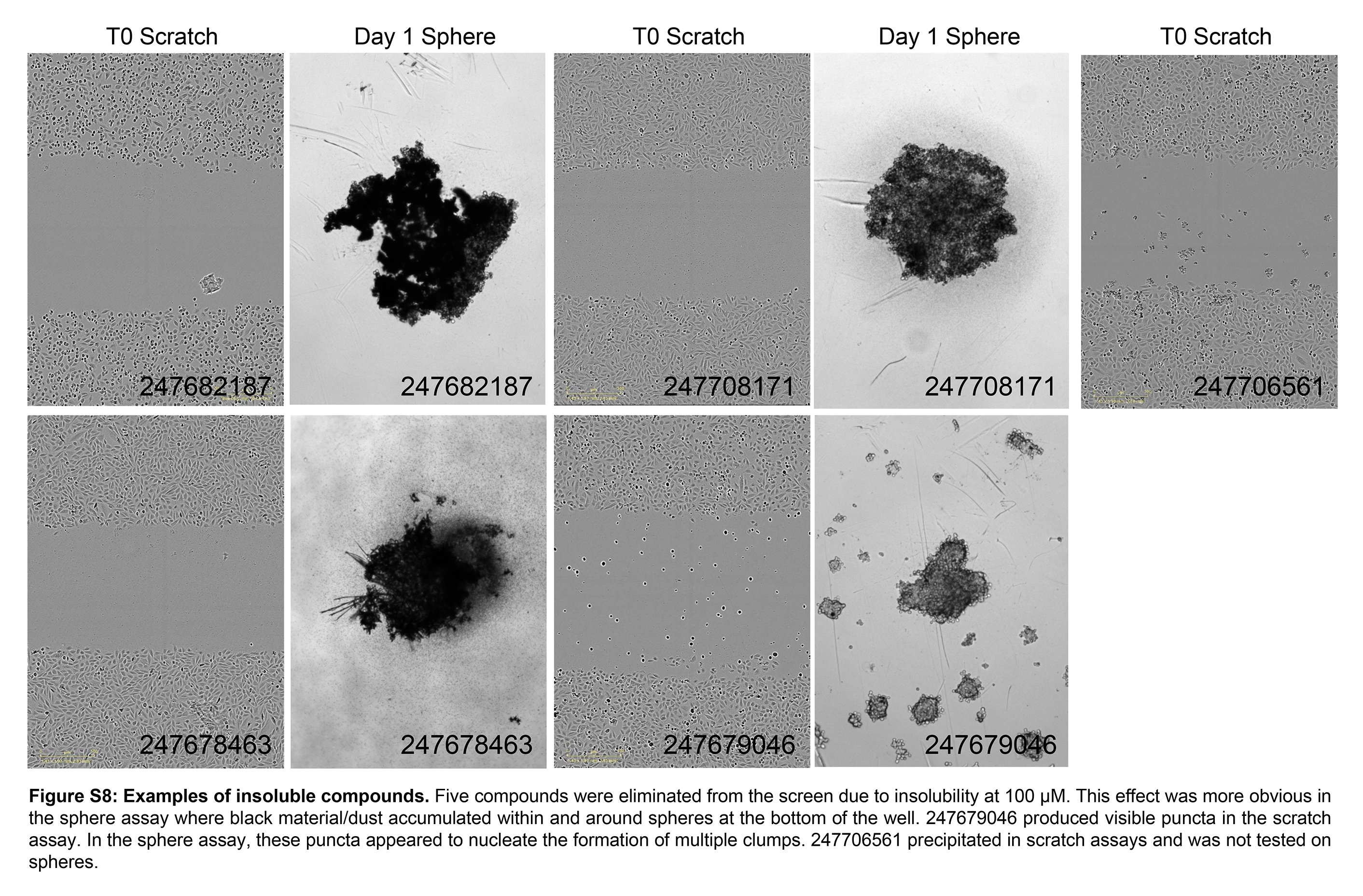

Supplement: Supplementary file 9 — Figure S8 [file JCMM-27-3553-s007.tif]

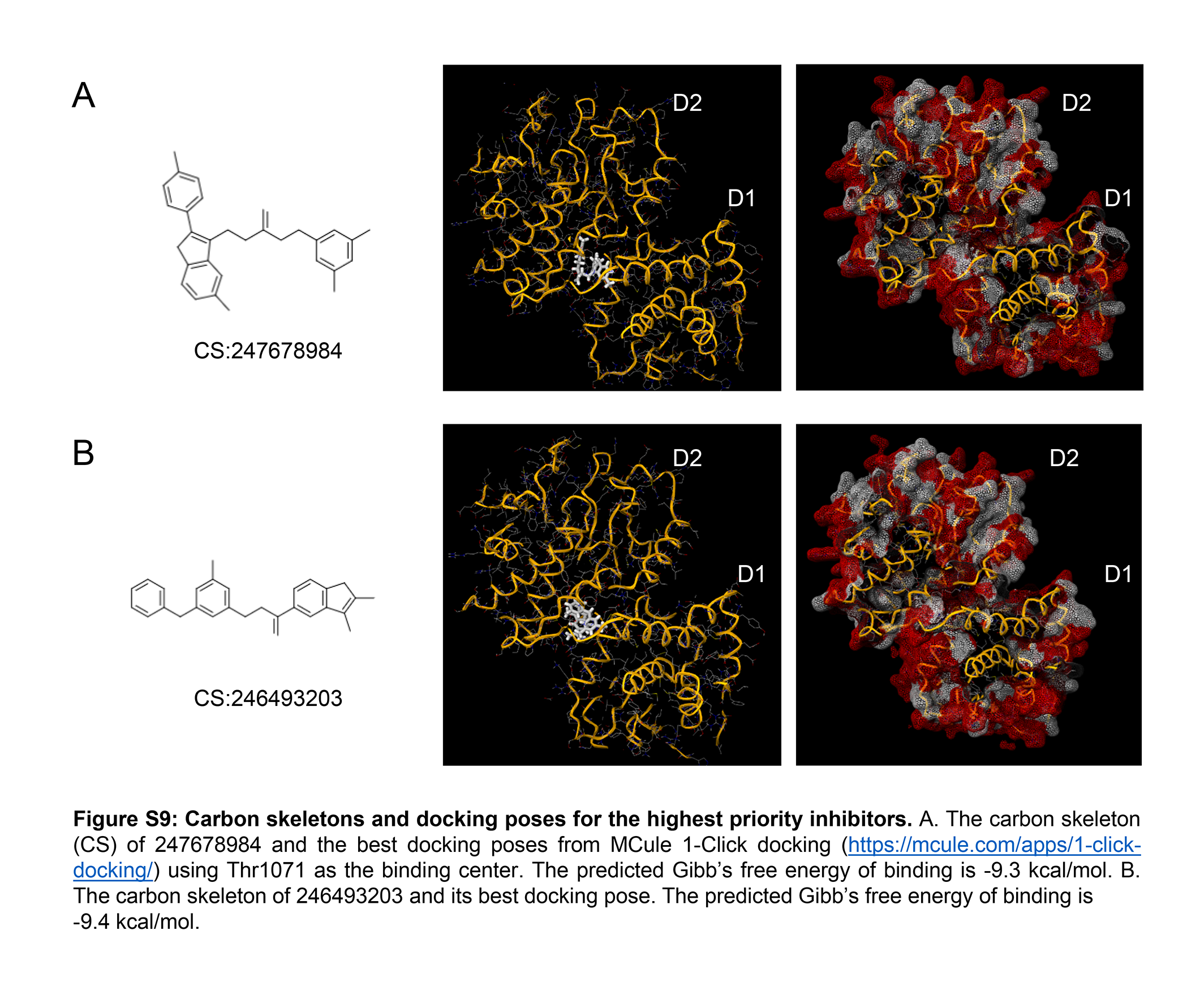

Supplement: Supplementary file 10 — Figure S9 [file JCMM-27-3553-s003.tif]
